# A next-generation sequencing approach for the detection of mixed species in canned tuna

**DOI:** 10.1016/j.fochx.2023.100560

**Published:** 2023-01-05

**Authors:** Regina Klapper, Amaya Velasco, Maik Döring, Ute Schröder, Carmen G. Sotelo, Erik Brinks, Marta Muñoz-Colmenero

**Affiliations:** a Max Rubner-Institut, Federal Research Institute of Nutrition and Food, National Reference Centre for Authentic Food, E.-C.-Baumann-Straße 20, 95326 Kulmbach, Germany; b Instituto de Investigaciones Marinas (CSIC), Eduardo Cabello 6, 36208 Vigo, Spain; c Max Rubner-Institut, Federal Research Institute of Nutrition and Food, Department of Safety and Quality of Milk and Fish Products, Palmaille 9, 22767 Hamburg, Germany; d Max Rubner-Institut, Federal Research Institute of Nutrition and Food, Department of Microbiology and Biotechnology, Hermann-Weigmann-Str. 1, 24103 Kiel, Germany

**Keywords:** Amplicon sequencing, *Thunnus* species identification, Seafood, Food fraud, NGS, Tuna cans

## Abstract

Tuna cans are relevant seafood products for which mixtures of different tuna species are not allowed according to European regulations. In order to support the prevention of food fraud and mislabelling, a next-generation sequencing methodology based on mitochondrial cytochrome *b* and control region markers has been tested. Analyses of defined mixtures of DNA, fresh tissue and canned tissue revealed a qualitative and, to some extent, semiquantitative identification of tuna species. While the choice of the bioinformatic pipeline had no influence in the results (p = 0.71), quantitative differences occurred depending on the treatment of the sample, marker, species, and mixture (p < 0.01). The results revealed that matrix-specific calibrators or normalization models should also be used in NGS. The method represents an important step towards a semiquantitative method for routine control of this analytically challenging food matrix. Tests of commercial samples uncovered mixed species in some cans, being not in compliance with EU regulations.

## Highlights

• A next-generation sequencing method for canned tuna is presented.
• The method detects different tuna species in mixtures.
• Amplification of mtDNA markers *cytb* and control region.
• Qualitative, and to some extent, semi-quantitative results.
• Different bioinformatic analyses lead to similar results.

## 1 Introduction

Tunas are in the top of the commercially most relevant seafood species worldwide with annual landings of 5.3 million tonnes in the year 2019 (Fao, 2020, Issf, 2021). The most important commercial tuna species are skipjack tuna (*Katsuwonus pelamis*, Linnaeus 1758) which accounts for about 60 % of the global catch, followed by yellowfin tuna (*Thunnus albacares*, Bonnaterre, 1788) with 28 %, bigeye tuna (*T. obesus*, Lowe, 1839) with 7 %, albacore tuna (*T. alalunga*, Bonnaterre, 1788) with 4 %, and Atlantic bluefin tuna (*T. thynnus*, Linnaeus, 1758) with 1 % (ISSF, 2021). Tunas are of high value, especially the species albacore or “Bonito del Norte” and Atlantic bluefin (Fao, 2020, Gordoa et al., 2017). Tunas are sold fresh, dried and frozen, but, on the European food market tuna cans are especially popular. For canning, the principal species used are skipjack and yellowfin tuna (Servusova & Piskata, 2021). The prices for canning vary among species, with the lowest market price for the most common species, skipjack and yellowfin tuna (FAO, 2020).

Intentional or unintentional substitution of tuna species may originate from the fact that they share very similar morphological characters, varying qualities and market values among species (Servusova & Piskata, 2021). In the European Union, Regulation EU 1379/2013 indicates mandatory information for the labelling of seafood products in general, in which, inter alia, commercial and scientific names need to be provided. Canned and other prepared products do not fall under the requirement of showing the scientific name and only the commercial name is mandatory. Regarding the labelling of canned tuna, the Council Regulation EEC 1536/92 states that preserved tuna and bonito requires only commercial names, but they must be prepared exclusively from one species while the mixing of species is not allowed unless the muscular structure has disappeared. In some European countries, state regulations are stricter, establishing which species can go under a certain commercial denomination on the label. For instance, in Spain, the RD 1385/2009 of August 28 establishes that under the commercial name “light tuna” (“atún claro” in Spanish) only yellowfin or bigeye can be canned, under the name of only “tuna” it can be any of the above-mentioned species or Atlantic bluefin or skipjack, while under the denomination of “white tuna” (“atún blanco” in Spanish) or “Bonito del Norte” only albacore can be canned. In Germany, a species indication is currently not mandatory, however, many producers voluntarily indicate the scientific name. Therefore, mislabelling in canned tuna can be caused both by species substitution or by the addition of a second species. Indeed, a research of Sotelo et al. (2018) found a 7.8 % mislabelling rate for canned tuna in European products. Half of the mislabelled cans labelled as yellowfin were identified as albacore. Servusova and Piskata (2021) also analysed canned tuna and found that 19.2 % of skipjack and 24.4 % of yellowfin cans were mislabelled, and one can was identified as a mix of yellowfin and skipjack.

In order to combat food fraud, controls need to be carried out for testing the authenticity of products. DNA-based methods are the primary choice of analysis when a morphological identification is not possible, as is the case for processed products. Sanger sequencing with barcode primers is the gold standard (Hellberg et al., 2016, Sanger et al., 1977). However, tuna pose a particular challenge in this analytical approach for various reasons. On the one hand, tuna species are phylogenetically closely related and therefore they show a high similarity between their DNA sequences (Vinas & Tudela, 2009). Another point concerns the cans, as the sterilization process during canning leads to strong DNA degradation with possible base pair substitutions and to a fragmentation into short DNA sequences (Pecoraro et al., 2020, Ram et al., 1996). Several studies focused on these issues, either by searching for suitable markers or by optimizing DNA extraction protocols (Mitchell and Hellberg, 2016, Roungchun et al., 2022, Vinas and Tudela, 2009). Common markers for the identification of tuna species are mitochondrial fragments of cytochrome *b (cytb)* (Bojolly et al., 2017, Espiñeira et al., 2009, Sotelo et al., 2018) or the control region (Mitchell and Hellberg, 2016, Vinas and Tudela, 2009). An advantage of the control region is that interspecies variability of sequences is larger than in other barcoding markers as *cytb* and cytochrome *c* oxidase subunit I (COI) (Roungchun et al., 2022).

Regarding the issue of mixed species in cans, Sanger sequencing is not applicable for mixed products due to an overlap of peaks in the nucleotide sequence. Real-time PCR is suitable for semiquantitative identification of species in mixtures depending on the food matrix and the target species, but can only detect a limited number of target species simultaneously (Bojolly et al., 2017). Next-generation sequencing is a method for massive parallel sequencing to overcome this problem and is considered as a promising tool for routine analysis for mixed products to control authenticity (Haynes et al., 2019, Szabo et al., 2020). This method is rather untargeted and can therefore identify a higher number of species that would be ignored in targeted approaches such as real-time PCR (Baetscher et al., 2021). Thus, NGS allows the identification of unexpected species in food products and several studies, especially metabarcoding methods, exist for the analyses of various foods (Baetscher et al., 2021, Piredda et al., 2022, Varunjikar et al., 2022). Metabarcoding by the use of universal primers is suitable for identifying a range of species, but in some cases cannot distinguish between closely related ones such as tuna. Kappel et al. (2017) analysed self-generated mixtures of tuna and tested some tuna cans as market samples through an NGS approach by using the two cytochrome *b* fragments. The results were promising; however, a bias was found primarily with skipjack being overrepresented. The determination of species proportions based on the number of NGS reads is known to be difficult and may result from different factors such as the species in the mixture or the processing degree of the samples (Dobrovolny et al., 2022).

The aim of this study was to follow up on the study of Kappel et al. (2017) in order to test I) an alternative primer combination targeting a mitochondrial cytochrome *b* and control region as well as to II) better characterize factors influencing the measured proportions of tuna species in mixtures by testing samples of different processing degree and analysing NGS reads by different bioinformatic pipelines. Further, the method was applied to commercial tuna can samples to examine the compliance with EU declaration regulations. The purpose of this study was to progress in the analytics of the difficult food matrix of tuna cans as well as to evaluate the possibility for standardisation in future.

## 2 Material and methods

### 2.1 Sampling

Common species in tuna cans were selected for this study: skipjack (Kpel), albacore (Tala), yellowfin (Talb) and bigeye (Tobe)*.* One individual of each species (whole specimens or fillets) was purchased from local suppliers from Vigo except of one from Germany. All tissue samples were stored at IIM-CSIC in Vigo (Spain) at −80 °C until processing. In addition, eight commercial tuna cans were purchased from local markets and supermarkets in Germany and Spain (see details in Table 2).

### 2.2 Experimental design

**Fig. 1:**
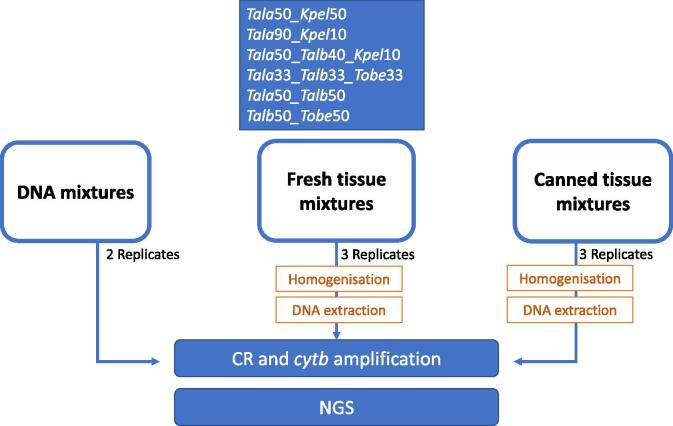
Experimental Design. Six mixtures were prepared for each treatment (DNA, FRE CAN). Skipjack (Kpel), albacore (Tala), yellowfin (Talb) and bigeye (Tobe). Tissue samples were homogenized and extracted. Amplicon fragments of BDR and CR were prepared for NGS.

The assay design is shown in Fig. 1. In order to test whether different treatments have an influence on the resulting proportion of reads recovered for each species in the mixture, mixtures of fresh samples (FRE), canned samples (CAN) as well as DNA mixtures (DNA) were prepared (for additional details on the preparation of the mixtures see Annex I A. The species mixtures prepared were: (1) Tala50_Kpel50, (2) Tala90_Kpel10, (3) Tala50_Talb40_Kpel10, (4) Tala33_Talb33_Tobe33, (5) Tala50_Talb50, and (6) Talb50_Tobe50 as DNA (volume per volume, duplicates), FRE (weight by weight, triplicate), and CAN (weight by weight, triplicate). These samples were tested with *cytb* (BDR) and control region (CR) markers and analysed the NGS data through six different bioinformatic pipelines.

### 2.3 DNA extraction

The DNA for all samples used in the mixtures (fresh tissue for DNA mixtures and lyophilised from fresh and canned mixtures, see details on lyophilisation in Annex I A) was extracted with the Wizard DNA Clean up system (Promega, Germany) at the IIM-CSIC (Spain). Briefly, a portion of 0.1 to 0.3 g of tissue was mixed with 860 µL of extraction buffer (1 % Sodium Dodecyl Sulfate (SDS), 150 mM NaCl, 2 mM Ethylenediaminetetraacetic acid (EDTA), and 10 mM Tris-HCl at pH 8, 100 µL of guanidinium thiocyanate 5 M and 40 µL of Proteinase K (20 mg / ml). After vortexing, samples were incubated for 2 h in a thermomixer at 56 °C and 800 rpm. After that time, another 40 µL of Proteinase K were added to each sample and the incubation continued overnight. Then, the protocol indicated by the manufacturer was followed for the isolation of the DNA with an elution volume of 50 µL. The DNA extraction from fresh and canned mixtures was performed in triplicate and the three isolated DNA tubes pooled into one.

For commercial cans, the Nucleospin Food kit (Macherey-Nagel, Germany) was used for the DNA extraction at the MRI (Germany). 13.75 mL buffer CF and 250 µL Proteinase K (10 mg/mL) were added to the 5 g homogenized sample material, vortexed and incubated at 65 °C overnight. The samples were centrifuged at 4500 rpm for 10 min. The clear supernatant was divided into aliquots of 1.5 mL reaction tubes and centrifuged at 14,000 rpm for 10 min. 400 µL supernatants of the aliquots were taken and the procedure was followed as described by the manufacturers. The elution of the DNA was performed using 50 µL CE buffer.

The preparation and DNA extraction of mixtures was done at IIM-CSIC (Spain) and DNA extraction of cans was done at MRI (Germany). The different DNA extraction methods were used due to the fact that the laboratories have different equipment and have different standard methods for DNA extraction. At both institutes, representative samples were taken for DNA extraction and both DNA extraction methods have been tested to perform well.

Double-stranded DNA was quantified using the Qubit dsDNA BR Assay Kit (Life Technologies, USA) for the tuna samples and with Qubit dsDNA HS Assay Kit (Life Technologies, USA) for the DNA of commercial cans due to a low DNA concentration of the latter extracts. Measurements were conducted on a Qubit 3.0 fluorometer (Invitrogen, USA). Purified DNA was stored at −20 °C until further analysis.

### 2.4 Primer design

In a first step, a literature research was conducted to find possible gene fragments suitable for tuna species differentiation. For the design of a new forward primer MH-Tuna-CR-V2 to shorten the CR fragment of Mitchell and Hellberg (2016), sequences used in Vinas and Tudela (2009) and further sequences from Genbank were aligned as references (see Annex I B). The analysis included sequences of the following *Thunnus* species: *T. albacares*, *T. alalunga*, *T. thynnus*, *T. orientalis*, *T. obesus*, *T. tonggol*, *T. maccoyii*. Additionally, sequences of *K. pelamis* were tested. CR as well as BDR primers were tested for the ability to be differentiated between species by performing a FINS analysis (see Annex I B Fig. a). %GC content, annealing temperature and self-dimerization calculations were performed in Oligocalc (http://biotools.nubic.northwestern.edu/OligoCalc.html). Amplification of a control region (CR) fragment using the new forward primer MH-Tuna-CR-V2 5′-GACATAYATGTATTAWAACCAT-3′ (this study) and reverse primers MH-Tuna-CR-R1 5′-CTGGTTGGTRGKCTCTTACTRCA −3′, MH-Tuna-CR-R2 5′-CTGGATGGTAGGYTCTTACTGCG −3′ (Mitchell & Hellberg, 2016) and a short *cytb* fragment with primers BDR-L 5′–GCMAACGGSGCNTCYTTCTTCT-3′ and BDR-H-mod1 5′-TGACGGTAGCHCCTCAGRADGACATTTGTCCYCA-3′ (unmodified and modified, respectively, according to González Sotelo, Medina, Pérez Martín, Quinteiro, & Rey Méndez (2002), as in Kappel et al. (2017), were also tested on fresh and canned products. Different annealing temperatures, number of PCR cyclers, MgCl_2_ concentrations, DNA concentration in the PCR reaction, and DNA polymerases were tested to optimize conditions for increasing the number of positive PCR amplification of tuna can samples for NGS.

### 2.5 Sanger sequencing

The four individuals used to make the mixtures were also sampled separately and analysed in all processing steps: DNA was extracted as in 2.3., amplified and sequenced in all stages (fresh, cooked, canned and lyophilized) with *cytb* (Burgener, 1997) and CR (this study) primers to monitor possible nucleotide substitutions due to processing. Sequencing was performed in an automatic ABI prism 3130 sequencer (Stab Vida LDA, Caparica, Portugal). For each marker and individual, sequences from all processing stages were aligned with Bioedit (Hall, 2011) and compared to check the presence of SNPs (Single Nucleotide Polymorphisms). The sequences obtained from the fresh samples were also used to confirm the species by FINS (Forensically Informative Nucleotide Sequencing) (Barlett and Davidson, 1992).

### 2.6 Amplicon sequencing NGS on the Illumina MiSeq platform

The two gene fragments were targeted, an approximately 170 bp fragment of the control region (CR) and a 131 bp *cytb* fragment (BDR). The sequencing was divided into two separated runs, one for the DNA-mixtures and some commercial tuna cans (TCA 36, 37, 38) using a MiSeq Reagent Micro Kit v2: 4 M Reads (300 Cycles) and a second run for fresh and canned tuna mixtures and commercial tuna cans (TCA 42, TCA43, LC1, LC2, LC3) with the MiSeq Reagent Kit v2: 15 M reads (300-cycle), both Illumina (San Diego, USA). The preparation of the CR- and *cytb*-targeted NGS approach was performed as a two-step protocol according to the 16S Metagenomic Sequencing Library Preparation Guide of Illumina (Illumina Inc., USA). A detailed description of the procedures is provided in Annex I C. In brief, the preparation consisted of two steps of PCR, an amplicon-PCR and an index-PCR, followed by library quantification and normalisation with the final library denaturing and sample loading.

### 2.7 Bioinformatic analysis

**Fig. 2:**
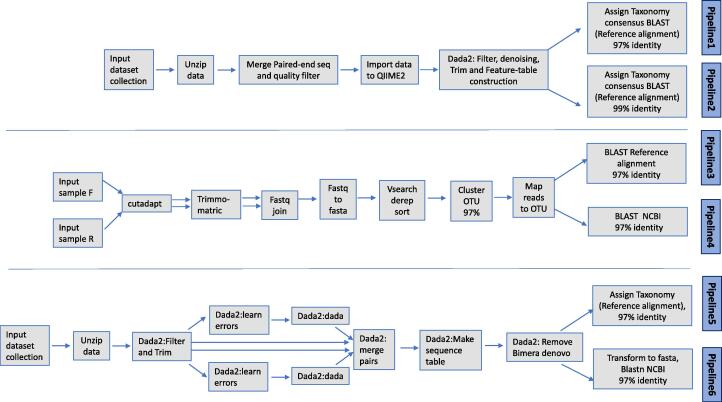
Scheme of the pipelines used to analyse the NGS data. Pipelines 1 and 2 were performed in CESGA using QIIME2 (Bolyen, et al., 2019), Pipelines 3–6 in Galaxy (Afgan, et al., 2018).

Indexes and adapters were automatically removed by the MiSeq software (Illumina). Six different bioinformatic analyses workflows were tested (Fig. 2). Two (with two versions for each one) were implemented as a Galaxy workflow (Afgan et al., 2018) and another one (with two versions), based mainly on QIIME2 v2021.4 plugins (Bolyen et al., 2019), was run using the servers of the “Centro de Supercomputación de Galicia” (CESGA). Custom databases were prepared and used for pipelines 1, 2, 3, and 5. In the case of BDR fragments, we developed our own database for scombroids with 258 sequences included in the IIM-CSIC in-house collection. For CR, the custom database (254 sequences) was formed by sequences from Genbank, used in Mitchell and Hellberg (2016), Viñas and Tudela (2009), and 36 additional sequences from the IIM-CSIC in-house collection.

Pipelines 1 and 2 (P1 and P2) correspond to the workflow run at CESGA. For both pipelines, forward and reverse reads were merged with minimum overlap of 15 nucleotides and without any mismatches, using the program PEAR Academic (Zhang et al., 2014). In this step, the sequences were also filtered by quality using Q20 as threshold. The filtered and merged reads were imported to QIIME2 via Manifest-file and a table of amplicon sequence variants (ASV) was constructed using dada2 plugin. This algorithm clusters the ASVs with 100 % of similarity, makes denoising, and removes the chimeras. The taxonomical assignment was performed using the consensus-blast plugin (Camacho et al., 2009), with 70 % of coverage and 97 % of identity for P1 and 99 % of identity for P2. The databases used for these two pipelines were the custom databases described above.

Pipelines 3 and 4 (P3 and P4) were implemented in Galaxy modified from Dobrovolny et al. (2019), see references for the tools therein. First, primers were removed using the Cutadapt tool, followed by Trimmomatic, using a minimum quality of 15 and 50 bp as minimum read length as requirements. Forward and reverse reads were joined, dereplicated, sorted and clustered into Operational taxonomic Units (OTUs), and mapped (Edgar, 2013). For P3, OTUs were compared against the custom databases mentioned above with at least 97 % identity. For P4, OTUs were compared against the NCBI nucleotide database (NCBI Resource Coordinators, 2014), by reporting the first BLAST hit within at least 97 % identity. OTUs with less similarity were declared as “no hits”.

Pipelines 5 and 6 (P5 and P6) used the Divisive Amplicon Denoising Algorithm Dada2 (Callahan et al., 2016), taking the ASV approach as described in Callahan et al. (2017). The workflow was implemented in Galaxy. The pipeline included the dada2 tools as recommended to turn paired-end fastq file into merged, denoised, chimera-free, inferred sample sequences (Callahan, et al., 2016, https://benjjneb.github.io/dada2/index.html). In the first step, complexity and quality profile plots were prepared. After the quality was estimated, the data was unzipped and primers were trimmed from the start and the end of the reads using the dada2 filter and trim tool and the quality was checked again. The reads were used to train for base-call error patterns in the dataset using the loess-error function and by applying dada to correct the trimmed reads. Forward and reverse reads were merged with a minimum overlap of 12 nucleotides and without any mismatches into a single sequence table. Chimeras were removed de novo from pooled samples. The sequence identification was performed by either aligning all inferred amplicon sequence variants to the custom databases of CR and BDR (P5) and to the NCBI nucleotide database (P6) and reporting the taxonomy assigned or the first BLAST hit within 97 % identity.

### 2.8 Statistical analyses

In order to eliminate potential of wrong species assignation due to errors in the amplification and sequencing process, a threshold of 1 % was set and the species detected in proportions lower than 1 % were removed. For the remaining assigned species, the frequencies were recalculated with respect to the new reduced total number of reads.

For the quantitative analysis, the cleaned data from the different approaches were integrated into a common data matrix including repeated measurements of the samples with respect to six pipelines (P1, …, P6), two markers (BDR, CR) and six recognized species (skipjack, albacore, yellowfin, bigeye, blackfin (*T. altanticus*) and bluefin).

The absolute difference of the observed proportion to the input proportion of species included in the mixtures (|o-t|) was used as input of a mixed effect model with pipeline, marker, treatment, mixture and species as fixed grouping factors. To model the repeated measurement of the same sample the sample-ID was used as random intercept effect. The influence of the fixed effects was evaluated by F-tests to the null-hypothesis of no differences between the theoretical means of their corresponding groups. To give each treatment equal weights the mean value of the observed proportion (m.o) over the two (DNA mixtures) or the three (fresh tissue mixtures and canned tissue mixtures) replicates were considered. The differences of the measured mean (with respect to the replicates) and the input proportions (m.o-t) were used to compare the accuracy of the pipelines visually as well as to check the trend of the species in the different mixtures (overestimation versus underestimation).

Subsequent statistical analyses were conducted exemplary for pipeline six. Mean and standard deviations of o-t are used to get an overview of the data. For each species 95 % confidence intervals for the expectation value of the difference of the measured proportion mean to the input proportion (m.o-t) were represented based on the *t*-distribution. Mean and standard deviations of |m.o-t| were used to compare the accuracy for different subsets of samples.

Separately for each species paired t-tests were performed to test the alternative hypothesis the true mean of the difference between the proportions by using BDR and CR marker is not equal to zero. Separately for each treatment the influence of the marker was compared by F-tests in mixed effect models with marker, mixture and species as fixed grouping factors and the sample-ID as random intercept effect.

All mentioned statistical analyses were performed with the software R (R Core Team, 2021) using the packages nlme (Pinheiro et al., 2021), ggplot2 (Wickham, 2016) and openxlsx (Schauberger & Walker, 2021). Statistical significance was set at the 0.05 level. However, due to the study design with its semiquantitative approach all significance statements are to be understood as explorative analysis.

## 3 Results

### 3.1 Sanger sequencing results

Forensically informative nucleotide sequencing (FINS) analysis of the CR fragment confirmed the *Thunnus* species used for making the artificial mixtures (data not shown). In the Sanger sequences of the *cytb* and the CR fragments from the different treatment stages (fresh, cooked, canned, and lyophilized), no nucleotide substitutions occurred in any species.

### 3.2 NGS qualitative results

The quality of sequencing was good. In run 1 the average number of reads per sample in the raw data from the sequencer (not taking the negative controls) was 128,258 for BDR and 170,205 reads for CR, except CR13 (CR, can from supermarket TCA36) which contained only 1,101 reads. In run 2 the average number of reads per samples was for BDR 140,707 and for CR 174,697, except for the sample BDR1-2F (BDR, FRE, Tala 50_Kpel50, replicate 3) which contained only few reads (1,348) and was excluded from the statistical analysis. The internal control of PhiX was sequenced correctly being 5.0 % of the total reads for the first run and 7.26 % for the second run. The read numbers of the negative controls, samples containing only water, were very low in comparison to the samples, as it was expected, with 4,916 (CR NTC, run1), 561 and 558 (CR NTC and BDR NTC run2 respectively). The negative controls contained mainly unassigned reads and albacore sequences, with minimal appearance of skipjack or yellowfin. The raw sequencing data have been deposited in the Sequence Read Archive (SRA) with links to BioProject accession number PRJNA854603 in the NCBI BioProject database.

After the analyses of the sample mixtures through all six bioinformatic pipelines (denoising, filtering, ASV table construction and taxonomical assignment), the species mainly detected were those included in the mixtures (Annex II). For CR samples additional species to those added in the mixtures were detected in low proportions (<1 %), principally from *Thunnus* genus or skipjack and exceptionally *S. sarda* in some fresh and canned samples analysed with pipeline 5. Since all non-target species appeared in low proportions, all of them were successfully removed after applying the 1 % threshold. In the case of the BDR samples, additional species (not included in the mixture) were also assigned in some samples which were not added to the mixtures, depending on the treatment, pipeline and whether they were blasted to a custom database or directly to the NCBI Genbank. When BDR was analysed with pipelines 1 and 2, only *Thunnus* species and skipjack (and *S. scombrus* in one canned sample) were additionally assigned in low proportions and removed after a 1 % threshold was applied. Only in one sample, Atlantic bluefin was detected > 1 %. Analysing with pipelines 3 and 4, in several samples Atlantic bluefin and blackfin (*T. atlanticus*) exceeded 1 %. In addition, other *Thunnus* species and skipjack were detected in low proportions with P3 and other non-scombroid species (*L. boscii, M. merluccius, S. surmuletus, P. maxima, S. rhombus, E. encrasicolus*) were also detected in low proportion with P4 (Annex II). All these species except of Atlantic bluefin and blackfin were removed after the 1 % threshold. Lately, after analyses with pipelines 5 and 6, non-target species (other *Thunnus* not included and skipjack for both, P5 and P6, *S. sierra* for P5 and *M. merluccius*, *P. maxima*, *L. boscii*, *S. rhombus* for P6) were only detected on low proportion and did not exceed 1 % threshold.

Summarizing, after removal of reads with <1 % of the total assigned reads of each sample, all CR samples included only reads assigned to species actually added to the mixtures. After the 1 % threshold in the BDR mixtures, besides the target species included in each mixture, Atlantic bluefin remained assigned in some samples (mixtures with Talb50_Tobe50 and Tala33_Talb33_Tobe33) after bioinformatic analyses with pipelines 1, 2, 3 and 4, with a maximum of 9.85 % in pipeline 3. Blackfin also remained after pipelines 3 and 4 in Tala50_Talb50, Tala50_Talb40_Kpel10 and Tala33_Talb33_Tobe33 mixtures, with a maximum of 6.99 %.

### 3.3 Pipeline comparisons

For the statistical analysis, some samples were excluded considered as outliers: BDR4-3F, CR4-3F – (BDR and CR, FRE, Tala33_Talb33_Tobe33, replicate 3) differed a lot from the remaining replicates probably due to a problem in the homogenization of the mixture; BDR9-3C – (BDR, CAN, Tala50_Talb40_Kpel10, replicate 3) and BDR11-3C – (BDR, CAN, Tala50_Talb50, replicate 3) probably due to pipetting errors.

The comparison of pipelines showed no significant effect (Mixed effect model |o-t|: Pipeline F = 0.58, p = 0.71). The differences between the observed mean and target proportions (m.o-t) is shown in Annex III: Figure. In order to reduce the complexity of the results and because the results of the pipelines were similar, we chose pipeline 6 as an example, from which we will show the results in the following sections.

### 3.4 Species and mixtures

**Fig. 3:**
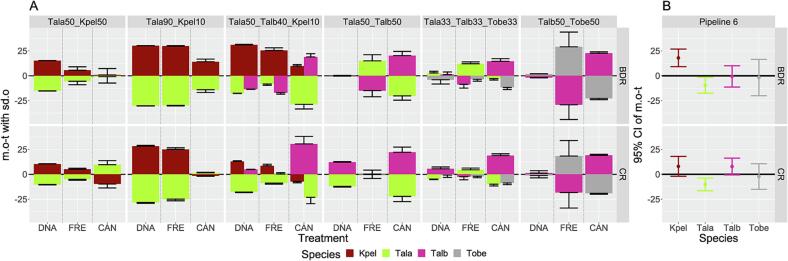
**A**. Mean and standard deviation of the difference between the observed values in the mixtures and the actual input per species in percent (y-axis) shown for different treatments (x-axis), markers (rows) and mixtures (columns). Skipjack (Kpel), albacore (Tala), yellowfin (Talb) and bigeye (Tobe). For a clear display, the standard deviations are shown one sided only. **B**. Measured deviations from input proportions per species and markers. Mean values with 95% confidence interval, Pipeline 6.

The results of the artificial mixtures from the three treatments (DNA, FRE, CAN) of pipeline 6 are summarized in Table 1 and shown in Fig. 3A. Overall, all species that were included in the artificial mixtures could be identified by each marker. Skipjack was overrepresented (lower 95 %-confidence limit for the expectation of m.o-t was 9.13, Fig. 3B), especially in mixtures with a proportion of only 10 % (Tala90_Kpel10 and Tala50_Talb40_Kpel10), which showed the largest m.o-t. There was only one exception in which skipjack was underestimated when the mixtures were prepared with canned tissue and amplified with CR. Proportions of skipjack in the cans were closer to the expected input proportion in all mixtures compared to the other treatments. Albacore was rather underrepresented in the samples and results of bigeye were the closest to the input proportions for both markers but with the highest standard deviation (Fig. 3B). Contrasting to the results of skipjack, yellowfin was more overrepresented in the cans in comparison to the other treatments and in CR, while with BDR the results were more variable.

**Table 1:** NGS results of the mixtures per DNA marker and treatment. For each sample, the mean and standard deviation of the two (DNA) or three (FRE, CAN) replicates are shown. Skipjack (Kpel), albacore (Tala), yellowfin (Talb) and bigeye (Tobe). Results of pipeline 6 hits with<1% and outliers were excluded from the analysis.

BDR	KPEL		TALA		TALB		TOBE	
*DNA_mixture*	Mean	SD	Mean	SD	Mean	SD	Mean	SD
Tala33_Talb33_Tobe33	0	0	36.14	1.64	34.52	2.63	29.33	4.27
Tala50_Kpel50	65.11	0.20	34.89	0.20	0	0	0	0
Tala50_Talb40_Kpel10	41.03	0.43	32.41	0.13	26.56	0.30	0	0
Tala50_Talb50	0	0	49.89	0.30	50.11	0.30	0	0
Tala90_Kpel10	40.15	0.04	59.85	0.04	0	0	0	0
Talb50_Tobe50	0	0	0	0	48.18	0.15	51.82	0.15

*Fresh_mixture*	Mean	SD	Mean	SD	Mean	SD	Mean	SD
Tala33_Talb33_Tobe33	0	0	45.46	1.83	24.71	3.72	29.84	1.89
Tala50_Kpel50	55.49	3.50	44.51	3.50	0	0	0	0
Tala50_Talb40_Kpel10	35.34	2.79	41.75	1.38	22.91	1.42	0	0
Tala50_Talb50	0	0	64.92	6.18	35.08	6.18	0	0
Tala90_Kpel10	39.78	0.50	60.22	0.50	0	0	0	0
Talb50_Tobe50	0	0	0	0	28.58	8.94	71.42	8.94

*Canned_mixture*	Mean	SD	Mean	SD	Mean	SD	Mean	SD
Tala33_Talb33_Tobe33	0	0	30.44	1.48	47.91	2.58	21.65	2.10
Tala50_Kpel50	50.86	6.43	49.14	6.43	0	0	0	0
Tala50_Talb40_Kpel10	19.74	1.49	21.33	4.67	58.92	3.17	0	0
Tala50_Talb50	0	0	37.62	13.95	62.38	13.95	0	0
Tala90_Kpel10	24.05	2.72	75.95	2.72	0	0	0	0
Talb50_Tobe50	0	0	0	0	72.63	1.36	27.37	1.36

CR	KPEL		TALA		TALB		TOBE	

*DNA_mixture*	Mean	SD	Mean	SD	Mean	SD	Mean	SD
Tala33_Talb33_Tobe33	0.00	0.00	28.59	0.57	38.87	1.79	32.54	2.36
Tala50_Kpel50	60.31	0.32	39.69	0.32	0.00	0.00	0.00	0.00
Tala50_Talb40_Kpel10	22.96	0.55	32.15	0.59	44.89	0.05	0.00	0.00
Tala50_Talb50	0.00	0.00	37.86	0.62	62.14	0.62	0.00	0.00
Tala90_Kpel10	38.26	0.87	61.74	0.87	0.00	0.00	0.00	0.00
Talb50_Tobe50	0.00	0.00	0.00	0.00	51.24	2.33	48.76	2.33

*Fresh_mixture*	Mean	SD	Mean	SD	Mean	SD	Mean	SD
Tala33_Talb33_Tobe33	0.00	0.00	37.85	1.76	30.87	3.10	31.28	1.34
Tala50_Kpel50	55.08	0.93	44.92	0.93	0.00	0.00	0.00	0.00
Tala50_Talb40_Kpel10	18.55	1.54	41.12	1.00	40.33	1.30	0.00	0.00
Tala50_Talb50	0.00	0.00	49.82	4.03	50.18	4.03	0.00	0.00
Tala90_Kpel10	34.98	1.63	65.02	1.63	0.00	0.00	0.00	0.00
Talb50_Tobe50	0.00	0.00	0.00	0.00	40.19	7.51	59.81	7.51

*Canned_mixture*	Mean	SD	Mean	SD	Mean	SD	Mean	SD
Tala33_Talb33_Tobe33	0.00	0.00	23.34	1.74	52.05	1.87	24.60	1.66
Tala50_Kpel50	40.32	4.04	59.68	4.04	0.00	0.00	0.00	0.00
Tala50_Talb40_Kpel10	2.02	0.28	23.33	2.49	74.65	2.76	0.00	0.00
Tala50_Talb50	0.00	0.00	27.85	5.19	72.15	5.19	0.00	0.00
Tala90_Kpel10	8.44	0.44	91.56	0.44	0.00	0.00	0.00	0.00
Talb50_Tobe50	0.00	0.00	0.00	0.00	69.06	0.94	30.94	0.94

### 3.5 DNA marker comparison

**Fig. 4:**
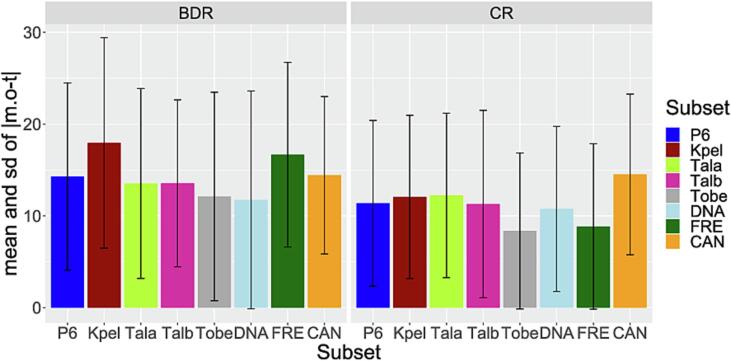
Mean and standard deviations of the absolute values of the differences between the mean of observed proportions and input proportions in the mixtures for BDR and CR. Skipjack (Kpel), albacore (Tala), yellowfin (Talb) and bigeye (Tobe). Results are shown for pipeline 6, per species, and per treatment.

In the comparison as to which marker gave the more accurate results in terms of quantification, CR results were slightly better than the BDR results (Fig. 4). The mean and standard deviation of |m.o-t| was lower for CR than for BDR (CR 11.38 ± 9.02, BDR 14.29 ± 10.2) and significant differences between the markers were found in the subset of all samples analysed with P6 (Mixed effect model, p-values of marker-differences P6 *p* < 0.01). This can mainly be attributed to differences in skipjack and yellowfin, since paired t-tests to the difference of the results between BDR and CR revealed significant results for skipjack (*p* < 0.01) and yellowfin (*p* < 0.01), while there were no significant differences for albacore (p = 0.71) and bigeye (p = 0.67) between the two markers. The measured results of the FRE samples were closer to experimental input proportions for the CR (Mixed effect model, p-values of marker-treatments DNA 0.14, FRE < 0.01, CAN 0.57).

### 3.6 Commercial cans

Eight commercial cans from German and Spanish supermarkets were analysed using the NGS approach (Table 2). Four cans labelled as albacore were identified as this species by both markers with 100 % of assigned reads, so no mixture with other species was detected. One can labelled as *Thunnus* sp. was identified as bigeye with 100 % of assigned reads by both markers. Two samples declared as “Atun claro” (light tuna) were found to include mixtures of other *Thunnus* species. In both cases a mixture of yellowfin with bigeye was detected by both markers. In sample LC2 (“Atun claro”) the results of the two markers were in accordance. In sample TCA36, the read number of the CR marker sample was too low to be analysed (715 reads) and this CR result was discarded for this sample, but the BDR reads clearly identified a mixture of yellowfin and bigeye. In sample TCA43 (*Katsuwonus pelamis*), the result was ambiguous: the CR marker identified a mixture of skipjack with albacore, while in BDR assigned reads of albacore were <1 % (after removing assigned reads with <1 % threshold).

**Table 2:** Analysis of commercial cans applying the NGS method. Origin and labelling of the samples and results as proportions of the assigned reads. “Label correct” refers to whether the label contains the expected species. Skipjack (Kpel), albacore (Tala), yellowfin (Talb) and bigeye (Tobe). Results of pipeline 6. Note, that no results are shown for the CR marker in the TCA36 as it only contained 1101 reads.

**Sample ID**	**Label**	**Origin**	**Price/can [€]**	**Declared species**	**Expected species**	**Marker**	**KPEL**	**TALA**	**TALB**	**TOBE**	**TTHY**	**Replicates**	**Label correct**
Tca36	Light tuna in olive oil	Hamburg, Germany	1.99	Atun claro	*T. albacares or T. obesus*	CR	NA	NA	NA	NA	NA	1	
						BDR	0.0	0.0	13.2	86.8	0.0	1	No
Tca37	White tuna in organic olive oil	Hamburg, Germany	5.69	*T. alalunga*	*T. alalunga*	CR	0.0	100.0	0.0	0.0	0.0	1	
						BDR	0.0	100.0	0.0	0.0	0.0	1	Yes
Tca38	Tuna fillets, white meat, in olive oil	Hamburg, Germany	4.79	*T. alalunga*	*T.alalunga*	CR	0.0	100.0	0.0	0.0	0.0	1	
						BDR	0.0	100.0	0.0	0.0	0.0	1	Yes
LC1	“Atún en aceite de girasol” (Tuna in sunflower oil)	Vigo, Spain	1.59	Atún (Tuna)	*Thunnus* sp. or *Katuwonus pelamis*	CR	0.0	0.0	0.0	100.0	0.0	2	
						BDR	0.0	0.0	0.0	100.0	0.0	2	Yes
LC2	“Atún claro en aceite de oliva“ (Light meat tuna in olive oil)	Vigo, Spain	1	Atún claro (Light tuna)	*T. albacares or T. obesus*	CR	0.0	0.0	72.86 ± 0,2	27.1 ± 0.2	0.0	2	
						BDR	0.0	0.0	80.5 ± 0.3	19.5 ± 0.3	0.0	2	No
LC3	“Bonito del norte en aceite de oliva” (White tuna in olive oil)	Vigo, Spain	1.54	Bonito del Norte (White tuna)	*T. alalunga*	CR	0.0	100.0	0.0	0.0	0.0	2	
						BDR	0.0	100.0	0.0	0.0	0.0	2	Yes
TCA42	White tuna in olive oil	Hamburg, Germany	2.99	Bonito del Norte (*T. alalunga*)	*T. alalunga*	CR	0.0	100.0	0.0	0.0	0.0	2	
						BDR	0.0	100.0	0.0	0.0	0.0	2	Yes
TCA43	Tuna fillets in sunflower oil	Hamburg, Germany	1.19	*Katsuwonus pelamis*	*K. pelamis*	CR	90.3 ± 1.8	9.7 ± 1.8	0.0	0.0	0.0	2	
						BDR	99.5 ± 0.7	0.5 ± 0.7	0.0	0.0	0.0	2	Ambiguous

## 4 Discussion

Tuna cans pose an enormous challenge for authenticity analysis due to the close phylogenetic relationships among tuna species and the high degree of processing of the product. Nevertheless, the control of correct labelling is essential in order to prevent or combat food fraud in these commercially very important and vulnerable species. The present study, an extension of the work of Kappel et al. (2017), leads to new insights regarding the applicability of markers and the influence of the treatment stages on quantitative assessments for mixed tuna cans. The study thus represents a progress towards the establishment of a semiquantitative method for authenticity control using next-generation sequencing.

### 4.1 NGS primer selection and Sanger results

For the development of a next-generation sequencing method to identify tuna species in mixed products, suitable gene markers had to be established. Considerations were (1) the ability to an unambiguous identification of the closely related tuna species most commonly used in cans and other scombroids using a short amplicon length due to the high DNA degradation in these products, (2) sufficient availability of reference sequences of all tuna species and further taxonomically close species to correctly align and assign the sequences, (3) and a constant successful amplification despite the high DNA degradation. After literature research, *in silico* analysis in Genbank (https://www.ncbi.nlm.nih.gov) and in BOLD (https://www.boldsystems.org), and some laboratory tests, the choice of primers for the NGS approach resulted in the two mitochondrial markers *cytb* and CR. The *cytb* BDR primers (González Sotelo et al., 2002, Mackie et al., 1999), modified in Kappel, et al. (2017), amplify a 131 bp fragment and have previously been shown to be suitable for tuna can analysis (Mariani et al., 2015). In laboratory tests, PCR amplification and sequencing results were positive for all tested species and thus proved the universal fit of the primers for tuna species DNA and the short length to amplify also degraded DNA in cans. For the CR primers, the reverse primer described by Mitchell and Hellberg (2016) were used, while the forward primer was newly designed. The reduced amplicon length of approximately 170 bp compared to the original 236 bp amplicon length (Mitchell & Hellberg, 2016) increased the rate for successful DNA amplification from degraded DNA in cans in laboratory tests. For the shortened DNA fragments of CR, FINS showed a differentiation of *Thunnus* species except for introgressed sequences of Atlantic bluefin and Pacific bluefin (*T. orientalis*) as well as Atlantic bluefin and albacore. The results were similar to the phylogenetic tree using mitochondrial control region sequences of Vinas & Tudela, (2009), in which introgressed mtDNA CR sequences of Atlantic bluefin and Pacific bluefin clustered to albacore and Atlantic bluefin, respectively (Annex I B). For the BDR fragment, *Thunnus* species can be differentiated except of the species blackfin and yellowfin as well as Atlantic bluefin and Southern bluefin (T*. maccoyii)* could not be differentiated by these primers. Even though the single DNA markers could not differentiate between all *Thunnus* species, the combination of these two markers (*cytb* and CR) could widely solve their differentiation. Low levels of introgression (2–3 %) are known from Pacific bluefin tuna and Atlantic bluefin, as well as from albacore DNA introgressed into Pacific bluefin or Atlantic bluefin (Alvarado Bremer et al., 1999, Alvarado Bremer et al., 2005, Vinas and Tudela, 2009). The introgression issue of albacore with Atlantic bluefin could be overcome by the use of the nuclear marker internal transcribed spacer I (ITS1), but this marker does not differentiate between Pacific bluefin and Atlantic bluefin (Mitchell & Hellberg, 2016). Within this study, despite large optimization efforts, tests with published (Mitchell and Hellberg, 2016, Vinas and Tudela, 2009) and modified primers of ITS1 were not satisfactory for this marker since the PCR amplification was not successful for all tuna species and many DNA extracts from cans (data not shown). This issue with ITS1 has been described in previous studies (Mitchell and Hellberg, 2016, Roungchun et al., 2022, Vinas and Tudela, 2009). The lack of reference sequences of all tuna and closely related species did not allow the design of primers for alternative DNA fragments. Therefore, CR and BDR were considered the best choice for our experiment.

Regarding the potential nucleotide substitution as a consequence of heat treatment, before the preparation of the mixtures, the sequences of each species were compared between each treatment (DNA, FRE, CAN). No differences were found, indicating no influence of the processing degree on the nucleotide sequence in our study. This finding is in contrast to Pecoraro, et al. (2020), where nucleotide substitutions were found after processing of tuna species, especially in cans after brine-canning operations and for yellowfin and skipjack (Pecoraro et al., 2020). The authors suggested these substitutions as source of potential misidentifications of canned specimens. In our case, with no differences among treatments, the misidentifications seem to be rather due to polymerase errors during amplification or sequencing, as well as to the bioinformatic pipeline and/or possible influence of the databases used in each case. However, we cannot discard that in industrial canning, processing can be even more aggressive due canning in brine than in our case and some modifications could somewhat increase the error rate in the identification.

### 4.2 NGS and pipelines comparison

All species that were added to the artificial mixtures could be identified and this applied to samples in all treatments. Thus, the method fulfils the intended purpose of detecting mixtures of tuna and identifying the species entered according to the Council Regulation EEC 1536/92. In both NGS runs, read numbers of negative controls were low and the reads that could be identified were mainly identified as albacore. The read number in negative controls can be explained either by slight contamination during the preparation of the samples for NGS analyses, sequencing errors in the indexes or by ‘index hopping’. The latter term describes an index mis-assignment between multiplexed libraries and the event increases when free adapters or primers occur in the NGS libraries (Guenay-Greunke et al., 2021). However, the numbers were within acceptable limits.

Comparing bioinformatic analyses, there were no significant differences, demonstrating the robustness of this methodology of identification with only subtle differences when distinct pipelines are applied. This is an important advantage since the bioinformatic pipeline used for NGS analyses is considered one of the most important sources of variability in the NGS studies (Siegwald et al., 2019, SoRelle et al., 2020, Walsh et al., 2018). Occasionally, species that were not added to the mixtures were found in certain samples, mostly in proportions lower than 1 %. Taking the low proportions of the assigned species that were not actually entered into our artificial mixtures into account, regardless of whether they are due to the algorithms used, sequencing errors, databases or impurities in the laboratory (Burns et al., 2016), we decided to use a threshold of 1 %. Additionally, in a metabarcoding study for seafood identification, taxa that make up > 1 % of fishmeal mixtures could consistently be detected, but rare taxa (<1 %) were detected inconsistently across markers and replicates (Baetscher et al., 2021), supporting our threshold set at 1 %. In the CR results, after applying the threshold, no species were found that were not actually added to the mixtures. In the BDR results, however, some other species were still identified, especially in Pip 3 and 4, but with mainly low proportions (9.85 % maximum). These false positive assignments were probably due to the different clustering algorithm used in Pip 3 and 4, based on OTU clustering instead ASV (Chiarello et al., 2022).

While in pipelines 1, 2, 3, and 5 sequences were blasted to a custom database, in pipelines 4 and 6, reads were blasted to the NCBI Genbank database. This had the advantage that it was not necessary to create an elaborated reference database with sufficient sequences that could potentially be expected in the tuna mixtures. A disadvantage may be that incorrect data entries will produce incorrect assignment results. Since there were no significant differences in the results of the six pipelines, we have presented the results of pipeline 6 as an example in the interests of clarity. Besides of not needing a custom database, another advantage of this pipelines was the use of Galaxy, since this platform is an online free platform to analyse NGS data and intuitive to use even for users with few bioinformatic skills (Afgan et al., 2018). It should be noted, however, that the other bioinformatics evaluation methods can be used equally well.

### 4.3 NGS markers specificity and accuracy and treatment effect

This experiment was designed to determine whether the NGS methodology can be applied as a semiquantitative approach. The results were satisfactory in terms of qualitative assessment and also a semiquantitative statement on the proportions of species in mixtures was possible in some cases. By using the BDR marker, a direct comparison to the findings of Kappel et al. (2017) was possible, through our fresh mixtures treatment, and these results were in accordance with the previous results. In both studies a clear overrepresentation of skipjack in comparison to *Thunnus* species was observed so this trend is maintained when BDR is used. While in the study of Kappel et al. (2017), the recovery of *Thunnus* species was similar for all tested species, with albacore and bigeye exceeding that of yellowfin, in our study albacore and yellowfin were rather underrepresented in the fresh mixture treatment, but yellowfin became dominant in the canned mixtures. The over-/underrepresentation maybe due to more copies of these markers could be present in skipjack mitochondrial DNA or DNA extraction is more efficient for this species/specimen.

Regarding the specificity of species identification in the mixtures, both mitochondrial markers were found to be suitable, however false-positive results occurred only in BDR results after applying the 1 % threshold for read proportions. Concerning the question which marker gave the more accurate results in terms of quantification, again CR results were slightly better than the BDR results. Given the shorter fragment length, the BDR primers have the advantage of a larger chance to successful amplification in samples with highly degraded DNA (Kappel et al., 2017). However, this short length and lower variability also seems to increase the risk of assignment errors. On the other hand, the selected markers in the present study were both mitochondrial markers. Mitochondrial markers are often used due to their high sensitivity and variability, but include the problem of a variable number of copies of mtDNA depending on the specimen, age, location and state of the extracted tissues limiting quantitative assessments (Bottero & Dalmasso, 2011). Besides the problem of introgression, this is another argument in favour of not to ceasing the search for adequate nuclear markers for the discrimination of tuna species in highly processed products.

An important finding from this experiment is that the deviation in proportions changed depending on the treatment (DNA, FRE or CAN). This was also different with respect to the marker used and type of mixture analysed. In the samples with skipjack included in low proportions (10 %), the overestimation of this species clearly destabilised the quantification. Surprisingly, this effect was lower in the case of canned products. It is known that the efficiency of the PCR is different depending on the matrix because the DNA degradation and potential inhibitors can hinder the amplification (Kim & Kim, 2019). Comparisons with other methods such as real-time PCR could also provide information about possible changes in primer efficiencies. Whether the results of these artificial mixtures are representative for the examined individuals of the four species must be shown in further tests but the direct comparison with the results of Kappel et al. (2017) indicates a certain consistency of the results for fresh tissue mixtures.

### 4.4 NGS as semiquantitative method to identify tuna mixtures

Alternative methods for semiquantitative identification of tuna mixtures are mainly real-time PCR assays. Methods for distinguishing yellowfin, Atlantic bluefin, albacore, or bigeye and Pacific bluefin, or skipjack and yellowfin focused on the species identification in the mixtures but neglecting the estimation of proportions (Chuang et al., 2012, Krčmář et al., 2019, Terio et al., 2010). Lopez and Pardo (2005) also performed tests for quantitative identification, in which the deviations between the real and calculated percentages from binary mixtures ranged from 0 to 25 % in tissue mixtures and ranged up to 50 % in sterilized tissue. In Bojolly et al. (2017) the proportions of Pacific bluefin / yellowfin could be partially achieved for Pacific bluefin, but the results for yellowfin were not conclusive when yellowfin tuna was added to > 50 % in mixtures. The available studies show that real-time PCR confronts similar issues in terms of quantification due to varying DNA concentrations, the difficult differentiation of tuna species and the high DNA degradation in processed products. In our case, except in three mixtures for BDR and two for CR, no deviation higher than 25 % in comparison to the expected proportion was obtained. By the application of the methodology presented here it is possible to detect the most commercially important *Thunnus* species and other closely related species with an acceptable approximation of real proportions in the majority of cases and no doubts about the presence of species mixture. NGS and especially metabarcoding has the advantage to potentially detect unexpected species (Preckel et al., 2021) and the power to analyse several samples at the same time, decreasing the price of analysis per sample (Haynes et al., 2019).

In the area of seafood identification, an increasing number of studies on metabarcoding approaches have been published in recent years, with focus mainly on qualitative identification of diverse species in processed surimi, fish products, and bivalve products in which thresholds for species detection ranged between 0.5 and 1 % (e.g. Baetscher et al., 2021, Gense et al., 2021, Giusti et al., 2019). Several NGS-based or metabarcoding methods also exist in the field of meat analysis (Ballin et al., 2009). A metabarcoding method for the identification of mammalian and poultry species in food (Dobrovolny et al., 2019) has already been tested for routine analysis (Preckel et al., 2021) and has been validated in an interlaboratory ring trial in order to harmonize analytical methods for food authentication (Dobrovolny et al., 2022). Based on the data of this ring trial, the authors suggested a threshold of 0.5 % to reliably assess the presence of a species in a food sample (Dobrovolny et al., 2022). However, this threshold is too small for our case, in which the species to discriminate are very closely related taxonomically. In this case, an analytical threshold of 1 % is more adequate as threshold in order to avoid errors due to similarity of sequences among the target species and the problems associated with the canning process, a matrix with particular analytical challenges (see also 4.2 and 4.3).

Regarding quantification, in meat analysis, the determination of the meat content of species is associated with similar problems, such as the use of mtDNA, processing grade and DNA extractability which impact on the quantitative results. Therefore, results are rather considered as rough estimates for the compositions of mixed species in food products (Cottenet et al., 2020, Dobrovolny et al., 2022, Preckel et al., 2021). Factors contributing to a bias of PCR-based methods can result from various sources in the analytical process. From sampling a bias can result from e.g. the fat content, the species, the effect of processing in the mitochondrial DNA, the muscle structure of species, or the number of mitochondrial genomes per cell. In the laboratory, sources for a bias are the DNA extractability, but also influences caused by the PCR itself like melting temperature of the strands, primer annealing to the target sequences or strand elongation in the PCR or NGS, the instrument and software, or the bioinformatics (Burns et al., 2016, Muñoz-Colmenero et al., 2021). The last one did not have a significant effect in our study. Despite of all these potential biases and the additional difficulty of tuna material, the results obtained with the methodology used here fit in line with previous works based on real-time tuna identification methods or NGS-based methods for detection of meat and seafood products, with good semiquantitative results able to discriminate the presence of mixtures of two or more species and a tentative proportion of them except those included in amounts ≤ 10 %.

For the use in routine analysis, further method validation including the determination of the amplification bias and the reproducibility for qualitative and quantitative application would be required. To improve the semiquantitative determination, the factors causing the observed deviations in the mixtures (treatments, species included in the mixtures) and the influence of the canning must be better characterised experimentally. This would also require testing several individuals of a species in order to determine if the intraspecific variation can modify the bias detected here and in Kappel et al. (2017). In this way, matrix-specific calibrators could later be used as reference standards, i.e., defining the bias present in mixtures of a similar degree of processing. This has already been applied in established real-time PCR methods, however, the production of these calibrators is rather complex (e.g., Köppel, Eugster, Ruf, & Rentsch, 2012). Alternatively, based on experimentally obtained data and the estimation of the deviations caused by the different factors (markers, species, mixtures, treatment), a normalisation method could be created, for example, by establishing mathematical models able to correct the differences in mitochondrial content, as well as, the different muscle structure of the species, in similar way to the models suggested to correct the variation in copy number of ribosomal markers (Darby et al., 2013, Lavrinienko et al., 2021).

### 4.5 NGS of commercial cans

In order to test the NGS method in practice, five cans from Hamburg, Germany, and three cans of Vigo, Spain, were purchased from supermarkets and analysed using the presented NGS approach. Mixtures of different species were detected from cans declared as “Atun claro” (Light tuna), one from Spain and one from Germany. In Spain, according to the RD 1385/2009, yellowfin or bigeye fall under the same trade name and are allowed to be canned as “light tuna” (“atún claro” in Spanish). In Germany, such a differentiation does not exist. However, in both cases according to EEC 1536/92 mixtures of different species may not be mixed in the same container. In both cases a mixture of yellowfin with bigeye could be detected. Yellowfin and bigeye are often mixed due to the simultaneous occurrence in schools leading to a combined capture in addition to a lack of different morphological characteristics at a small size (Bartlett and Davidson, 1991, Gordoa et al., 2017, Sotelo et al., 2018).

One sample of canned skipjack (TCA43) was ambiguous: While the CR results identified a mixture with albacore, only traces with<1 % were found in the results using the BDR primers. Compared to the other commercial can samples, a high number of different read sequences were found in this sample. A possibility may be issues during amplification and/or sequencing. This may also be a consequence of a high DNA degradation during the canning process and may as well lead to different quantitative results in the BDR and CR reads, e.g., if nucleotide substitutions occurred in the primer regions due to the more aggressive industrial canning processing. However, since we found albacore in both markers and replicates in TCA43, it can be assumed that at least traces of the species were present in the can. Since albacore has a higher market value than skipjack (FAO, 2020), a contamination of the processing facility would be more likely rather than a deliberate substitution (Sotelo et al., 2018). In Germany, the other way round is common since tuna cans of albacore are frequently substituted by less valuable skipjack (Kappel & Schröder, 2015).

The tests of commercial cans revealed the presence of mixtures of different species in some cans, which was not in compliance with EEC 1536/92. In routine analysis of meat products, undeclared proportions of <1 % (w/w) commonly occur but are usually not considered as violation of declaration (Cottenet et al., 2020). Regarding the distinction between adventitious contamination and deliberate substitution, contamination due to inadequate cleaning between processing batches should not exceed 5 % (w/w) in the case of meat (Cottenet et al., 2020, Defra., 2014, Waiblinger et al., 2017). Since unintentional carry-over in factories and the measurement uncertainties of PCR-based methods, particularly pronounced in these highly processed products should be accounted for, we propose a threshold of 5 % in the framework of this study and in conformity with practices in the food control as limit of unintentional proportions that could be found in tuna products without being understood as fraud.

## 5 Conclusions

NGS is a promising method for a broad application in food authenticity control and is of growing importance in routine analysis. In the present study, a NGS-method based on the amplification of two mitochondrial markers demonstrated the suitability to identify mixed tuna species through experiments on artificial mixtures. Tuna species could be identified from all mixtures at all processing stages and admixtures could be detected semiquantitatively to some extent. The use of the control region in addition to cytochrome *b* has been proven valuable. However, to distinguish between introgressed individuals and for improved quantification, future research focusing on the development of nuclear markers should be encouraged. The results of this study will support further progress towards a harmonisation and standardisation in the area of NGS analysis for tuna can authenticity by providing new insights into the reproducibility of results and the description of factors leading to deviations in quantitative results. For routine analysis on authenticity, further method validation and standardization would be required. The present study represents an important step towards the semiquantitative identification of the analytically challenging food matrix of tuna cans.

## Declaration of Competing Interest

The authors declare that they have no known competing financial interests or personal relationships that could have appeared to influence the work reported in this paper.

## Data Availability

The raw sequencing data have been deposited in the Sequence Read Archive (SRA) with links to BioProject accession number PRJNA854603 in the NCBI BioProject database

## Appendix A Supplementary data

Annex I Material and Methods details.

Annex II NGS results without threshold.

Annex III Figure. Difference between the observed mean and the target proportions (m.o-t) in percent (y-axis) shown for different pipelines (x-axis), markers (rows), mixtures (columns), treatments (shapes) and species (colour). Skipjack (Kpel), albacore (Tala), yellowfin (Talb) and bigeye (Tobe).
