# Supplementary material for: A next-generation sequencing approach for the detection of mixed species in canned tuna
Source: Food Chem X. 2023 Jan 5;17:100560. doi: 10.1016/j.fochx.2023.100560 (PMC9943852; doi:10.1016/j.fochx.2023.100560)
Supplement: Annex I — Material and Methods details. [file mmc1.docx]

**Annex I Material and Methods details**

1. Sample processing

These mixtures were made for the three different types of treatments following the next procedure:

- 1. DNA mixtures (DNA):

DNA from each individual was extracted with Wizard DNA Clean up system (Promega), following the manufacturer’s protocol. Isolated DNA was quantified with Qubit fluorometer 3.0 (Life Technologies) and Qubit dsDNA BR assay kit (Thermofisher Scientific) and then diluted to 50 ng/µL. The diluted DNA was mixed in the previously mentioned proportions to obtain 30 µL (1500 ng total) of each DNA mixture. Each mixture was made in duplicate.

- 1. Mixtures of fresh tissue (FRE):

Tuna tissue was allowed to thaw at 4 °C and was minced with a scalpel. Mixtures with a total weight of 30 g were made in triplicate in conical centrifuge tubes.

- 1. Mixtures of canned tissue (CAN):

After thawing at 4 °C, samples were cut into pieces of approximately 300 g and steamed (each species separately) in a Thermomix TM31 (Vorwerk) for 15 minutes at 99 °C. After steaming, the interior temperature of the pieces was measured in order to check that they were fully cooked (internal temperature ≥ 60 °C). Then, the samples were allowed to cool until they reached room temperature (20-30 °C), and this cooked tissue was used for making the mixtures of 30 g of total weight. For the canning procedure, the different sample mixtures were introduced in canning jars, adding salt (1 % in weight) and filling to the brim with sunflower oil. Jars were sterilized in an autoclave (Steam sterilizer, Trade Raypa) at 115 °C and 103 KPa, for 30 minutes. All mixtures were canned in triplicate. Sterilized jars were allowed to cool at room temperature and the oil was drained by inversion with a paper filter and the tissue was transferred to conical centrifuge tubes.

Fresh and canned mixtures were lyophilised in a BenchTop Pro3L XL-75 (SP Scientific), transferred to plastic bags and homogenized with a rolling pin. From each homogenized sample, three subsamples (replicates) of approximately 100 mg were separated for DNA extraction.

In the case of the commercial cans, oil was removed from the samples by squeezing out of the oil/water from the can and carefully removing the remaining liquid with paper towels. The whole tuna tissue was homogenized with pestle and mortar and three sub-samples of approximately 5 g were randomly taken and placed in 50 ml centrifuge tubes.

1. Primer development

**Sequences for primer development**

Table I. Sequences used for the development of the MH-Tuna-CR-V2 primer in combination with the reverse primers of Mitchell and Hellberg (2016).

| Species | GenBank Accession Numbers |
| --- | --- |
| *T. albacares* (Yellowfin tuna) | DQ126342.1 - DQ126345.1, KM261649.1 - KM261658.1, JN988640.1 - JN988644.1, KJ535780.1 - KJ535783.1, AB906809.1 |
| *T. thynnus* (Atlantic bluefin tuna) | AY650409.1 - AY650414.1AF390430.1, AF390432.1 - AF390439.1, KJ535775.1, KJ535776.1, KY491024.1 - KY491027.1, KR337440.1 - KR337442.1 |
| Pacific-like *T. thynnus* (Atlantic bluefin tuna) | AY650737.1, AY699944.1, AY650619.1, AY650594.1 |
| Albacore-like *T. thynnus* (Atlantic bluefin tuna) | DQ087593.1, DQ087541.1, AY650425.1, AF390425.1, AF390384.1 |
| *T. atlanticus* (Blackfin tuna) | MF416079.1- MF416083.1 |
| *T. tonggol* (Longtail tuna) | MF593039.1 - MF593048.1, MF593021.1 - MF593025.1, MF593012.1, MF593010.1 |
| *T. obesus* (Bigeye tuna) | HM572619.1, KM872020.1 - KM872030.1, KM261661.1 - KM261664.1, JN988647.1 - JN988649.1, AY640301.1 - AY640303.1 |
| *T. maccoyii* (Southern bluefin tuna) | MF416084.1 - MF416086.1, HQ630707.1, AB906819.1 |
| *T. orientalis* (Pacific bluefin tuna) | AB185022.1, HQ630709.1, AB536204.1 - AB536205.1, AB536248.1, AB536249.1, AB536271.1, AB536188.1 - AB536190.1, AB535859.1, AB535855.1, AB535856.1, AB535767.1, AB535768.1 |
| Atlantic-like *T. orientalis* (Pacific bluefin tuna) | GU972554.1 - GU972555.1, AB536503.1, AB536504.1 |
| *T. alalunga* (Albacore tuna) | MF416069.1, KP412826.1, AF390345.1 - AF390353.1, KJ535771.1, KJ535765.1, KJ535744.1, MF416077.1, MF416000.1 - MF416003.1, MF415933.1, MF415934.1, MF415974.1, MF415961.1, MF415963.1, MF415969.1, MF415970.1, MF415995.1 |
| *K. pelamis* (Skipjack tuna) | AB907528.1, JF752236.1, JF752048.1, JF752161.1, JF752079.1, JF752181.1, JF752083.1, KP669018.1, KP669081.1, KP938270.1, KM094155.1, KM094163.1, KJ617138.1, KJ617139.1, KJ617147.1, KJ617157.1, KJ617190.1 |

Table II. FINS results of CR fragment using the MH-Tuna-CR-V2 primer and reverse primers of Mitchell and Hellberg (2016)

| *T. albacares* | Differentiated by FINS |
| --- | --- |
| *T. tonggol* | Differentiated by FINS |
| *T. thynnus/ T. orientalis* | Introgressed sequences not differentiable by FINS |
| *T. obesus* | Differentiated by FINS |
| *T. alalunga/ T. thynnus* | Introgressed sequences not differentiable by FINS |
| *T. maccoyii* | Differentiated by FINS |
| *T. atlanticus* | Differentiated by FINS |
| *T. maccoyii* | Differentiated by FINS (only few sequences in Genbank) |

Table III. FINS results of the BDR fragment.

| *T. tonggol* | Differentiated by FINS |
| --- | --- |
| *T. obesus* | Differentiated by FINS |
| *T. alalunga* | Differentiated by FINS |
| *T. orientalis* | Differentiated by FINS |
| *K. pelamis* | Differentiated by FINS |
| *T. atlanticus/ T. albacares* | No differences between them |
| *T.thynnus/T. maccoyii* | No differences between them |


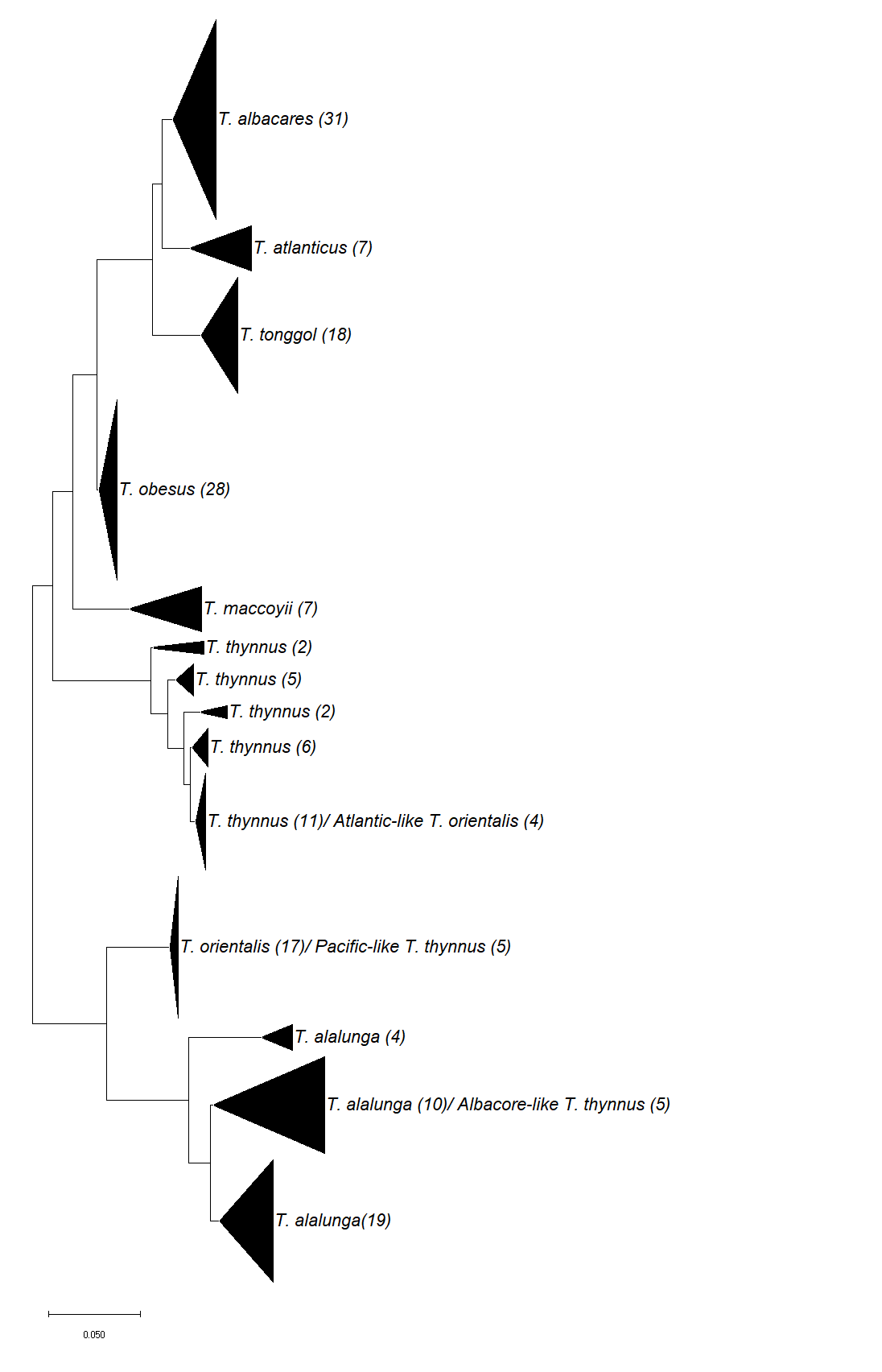


Fig. a. NJ tree of the shortened CR fragment of the present study using the Kimura 2-paramter model. Phylogenetic tree for FINS analysis using 181 Sequences of *Thunnus* species. The number of sequences in the branches provided in parentheses. Sequences included introgressed mtDNA CR sequences of *T. thynnus* and *T. orientalis* and *T. thynnus* and *T. alalunga* as presented in Vinas and Tudela (2009), additional sequences listed in Annex I Table I and own sequences. Tree is rooted at midpoint.

1. Details on Amplicon sequencing NGS on the Illumina MiSeq platform

The two gene fragments were targeted, an approximately 170 bp fragment of the control region (CR) and a 131 bp *cytb* fragment (BDR). The preparation of the CR- and *cytb*-targeted NGS approach was performed as a two-step protocol according to the 16S Metagenomic Sequencing Library Preparation Guide of Illumina (Illumina Inc., San Diego, CA, USA).

In the first step, PCR amplicons were prepared using primers containing the Illumina-specific adapters (synthesized by Biomers, Ulm, Germany). Samples were adjusted to 5 ng/µL and amplified with CR and BDR primers in a PCR with a final volume of 25 µL containing: 12.5 µL 2x Q5 HotStart Mix (NEB, Frankfurt, Germany), 0.5 pmol/µL of each primer and 10 µL DNA (5 ng/µL). The PCR was performed under the following conditions: for CR 98 °C initial denaturation 2 min, 30 cycles of denaturation 10 sec at 98 °C, annealing 30 sec at 49 °C, elongation 30 sec at 72 °C and a final elongation for 2 min at 72 °C. For BDR the protocol was the same except for the annealing temperature at 60 °C. A non-template control was added to each PCR run. Amplicons were checked on 2 % (w/v) agarose gels, followed with a purification of the PCR products using AMPure XP beads (Agencourt, USA). In brief, 20 µL of PCR product and 40 µL AMPure Beats solution were mixed in a MIDI well plate, placed on a magnet adapter, the supernatant was removed, and the product was washed twice with 80 % EtOH, dried on a magnet adapter. Then, 53 µL of 10 mM Tris pH 8.5 were added and 50 µL of the supernatant was recovered.

In the second step of the protocol, the indexed library was prepared. Each reaction had a volume of 50 µL including 25 µL Ultra II Q5 Master Mix (NEB), 5 µL of Nextera XT forward (29 bp) and Nextera XT reverse (24 bp), both Nextera XT v2 Index kit Set A (Illumina) and 5 µL of the purified PCR product. The Index-PCR cycler program was: 3 min initial denaturation at 95 °C, followed by 8 cycles of 30 sec denaturation at 95 °C, 30 sec annealing at 55 °C, 30 sec elongation at 72 °C, and a final elongation of 5 min at 72 °C. The resultant indexed-PCR products were checked for amplification and correct size again on 2 % (w/v) agarose gels. In a second purification step, 50 µL of indexed-PCR product was mixed with 56 µL AMPure XP beads and the procedure was repeated as in the first step. 28 µL of resuspension buffer (Illumina) were used to extract 25 µL of purified product. The DNA concentration of the purified products was then measured using the Qubit dsDNA Broad Range kit on a TECAN Spark 10 M microplate reader (TECAN, Männedorf, Switzerland). DNA concentrations were converted to nM with the formula *DNA concentration (ng/µL)/(660 g/mol* average DNA fragment size (bp))* 10^6^*, assuming that the approximate library size for CR fragments was 340 bp, and for BDR fragments 301 bp. DNA concentrations were adjusted to 4 nM using resuspension buffer (Illumina). The concentration was checked again using the Qubit dsDNA BR kit as described above. Normalized DNA libraries were combined to an 8 pM library pool. This library was denatured and 30 µL were loaded into Miseq mixed with 30 µL of 12.5 pM PhiX-control solution and pipetted into the cartridge (300-cycle MiSeq Reagent kit v2, Illumina).

References

Mitchell, J. K., & Hellberg, R. S. (2016). Use of the mitochondrial control region as a potential DNA mini-barcoding target for the identification of canned tuna species. *Food Analytical Methods*, *9*(10), 2711-2720.

Vinas, J., & Tudela, S. (2009). A validated methodology for genetic identification of tuna species (genus *Thunnus*). *PloS one*, *4*(10), e7606.
