# Supplementary figures and images for: A next-generation sequencing approach for the detection of mixed species in canned tuna
Source: Food Chem X. 2023 Jan 5;17:100560. doi: 10.1016/j.fochx.2023.100560 (PMC9943852; doi:10.1016/j.fochx.2023.100560)

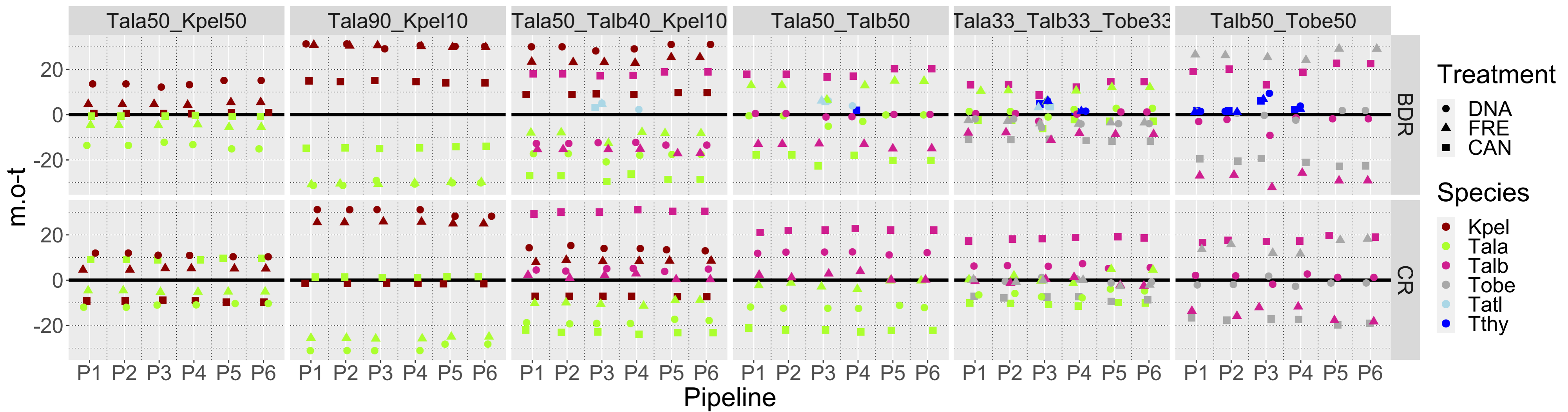

Supplement: Annex III — Figure. Difference between the observed mean and the target proportions (m.o-t) in percent (y-axis) shown for different pipelines (x-axis), markers (rows), mixtures (columns), treatments (shapes) and species (colour). Skipjack (Kpel), albacore (Tala), yellowfin (Talb) and bigeye (Tobe). [file mmc3.pdf]
